# The Host Factor Erlin-1 is Required for Efficient Hepatitis C Virus Infection

**DOI:** 10.3390/cells8121555

**Published:** 2019-12-02

**Authors:** Christina Whitten-Bauer, Josan Chung, Andoni Gómez-Moreno, Pilar Gomollón-Zueco, Michael D. Huber, Larry Gerace, Urtzi Garaigorta

**Affiliations:** 1 Department of Immunology and Microbial Sciences, The Scripps Research Institute, La Jolla, CA 92037, USA; 2 Department of Molecular and Cellular Biology, Centro Nacional de Biotecnología Consejo Superior de Investigaciones Científicas (CNB-CSIC), 28049 Madrid, Spain; 3 Department of Molecular Medicine, The Scripps Research Institute, La Jolla, CA 92037, USA

**Keywords:** hepatitis C virus, HCV, erlin-1, erlin-2, host factor, endoplasmic reticulum, RNA replication, protein production, virus production, lipid droplet

## Abstract

Development of hepatitis C virus (HCV) infection cell culture systems has permitted the identification of cellular factors that regulate the HCV life cycle. Some of these cellular factors affect steps in the viral life cycle that are tightly associated with intracellular membranes derived from the endoplasmic reticulum (ER). Here, we describe the discovery of erlin-1 protein as a cellular factor that regulates HCV infection. Erlin-1 is a cholesterol-binding protein located in detergent-resistant membranes within the ER. It is implicated in cholesterol homeostasis and the ER-associated degradation pathway. Silencing of erlin-1 protein expression by siRNA led to decreased infection efficiency characterized by reduction in intracellular RNA accumulation, HCV protein expression and virus production. Mechanistic studies revealed that erlin-1 protein is required early in the infection, downstream of cell entry and primary translation, specifically to initiate RNA replication, and later in the infection to support infectious virus production. This study identifies erlin-1 protein as an important cellular factor regulating HCV infection.

## 1. Introduction

Hepatitis C virus (HCV) is an enveloped virus belonging to the Flaviviridae family [1]. It is estimated that around 71 million people are chronically infected, many of whom will develop cirrhosis, end-stage liver disease and hepatocellular carcinoma [2].

The HCV genome is around 9.6 kb positive-sensed single-stranded RNA containing a unique long open reading frame (ORF) that encodes a single polyprotein of approximately 3000 amino acids [3]. The 5′ and 3′ nontranslated regions (NTR), flanking the ORF, contain essential sequences for RNA stability, translation and replication [4,5,6]. A highly structured internal ribosomal entry site located in the 5′ NTR drives the translation of the polyprotein that is co- and post-translationally processed by both host and viral proteases leading to the expression of three structural (core, E1 and E2) and seven non-structural proteins (p7, NS2, NS3, NS4A, NS4B, NS5A and NS5B) [7]. 

HCV entry into hepatocytes starts with the interaction of viral and cellular factors, present in the viral membrane, with several receptors present in the plasma membrane of the hepatocytes (reviewed in [8]). These interactions trigger the internalization of HCV via a receptor-mediated endocytosis process that is followed by the fusion of viral and cellular membranes and the release of the viral genome into the cytosol [9]. The incoming HCV RNA is then transported to intracellular membranes derived from the endoplasmic reticulum (ER) where primary translation and polyprotein processing occur. The viral replicase complex together with cellular factors is responsible for the RNA replication process, which starts by the generation of negative-sense RNA intermediates that are used as templates for the production of new positive-sense RNA molecules. Newly synthesized HCV genomes are then used as templates for translation, more rounds of RNA replication or packaging into progeny virus.

Lipid droplets (LD) have emerged as an important platform for HCV virus assembly [10], which is a highly coordinated process with several intermediate steps including: recruitment of NS5A and core proteins into LDs, encapsidation of HCV RNA into core particles and membrane envelopment of HCV RNA-containing core particles [11,12,13,14,15]. After budding into the ER lumen HCV exits the cell through the cellular secretory pathway. Given the tight association to the ER throughout different steps in its life cycle, it is not surprising that several ER and ER-related proteins have been identified as host factors regulating the HCV infection e.g., signal peptide peptidase [16], Sigma-1 receptor [17].

The erlin proteins, erlin-1 and erlin-2, are endoplasmic reticulum membrane lipid raft-associated proteins that belong to a larger family of proteins containing a conserved stomatin, prohibitin, flotillin, HflK/C (SPFH) domain which is proposed to organize membrane microdomains [18,19,20]. Both erlins are closely related as they share ~80% identity at the amino acid level [20,21] and they are evolutionarily conserved with homologous proteins found in *Caenorhabditis elegans* and *Arabidopsis thaliana* [21]. Erlin proteins are located in detergent resistant membranes (DRM) where they form high molecular weight complexes containing erlin homo- and hetero-oligomers as well as other cellular proteins [22]. Early reports described the function of erlin-1 and erlin-2 proteins in the endoplasmic reticulum associated degradation (ERAD) of inositol 1,4,5-triphosphate (IP3) receptors (IP3Rs) [23,24]. Afterwards other reports suggested that erlin-2 protein is required for the sterol-induced degradation of cholesterol biosynthetic enzyme HMG-CoA reductase [25] and for the processing of amyloid β-peptide (Aβ) precursor (APP) into Aβ by γ-secretase in the brain [26]. Besides their function in the ERAD pathway erlin proteins have been shown to regulate cholesterol homeostasis. They are cholesterol-binding proteins that interact with the sterol regulatory element binding protein (SREBP)-Scap-Insig complex restricting SREBP activation and leading to an intracellular accumulation of lipids and cholesterol [27]. More recently, Inoue and Tsai reported the first link between erlin proteins and viral infections [28]. They showed that erlin 1 and erlin 2 proteins are both required for polyomavirus SV40 infection by facilitating B12 transmembrane J-protein mobilization to specific foci in the ER, a prerequisite for the ER to cytosol transport of SV40, thus enabling the establishment of infection [28].

In view of the cellular functions and ER localization of erlin proteins, and considering the dependence of HCV on lipid metabolism and the ER for its life cycle, we decided to investigate the potential role of erlin proteins in HCV infection. In this study we describe the discovery that erlin-1 protein regulates the initiation of HCV RNA replication, the accumulation of viral proteins and therefore, the production of infectious virus, adding erlin-1 to the list of host factors required for efficient HCV infection.

## 2. Materials and Methods

### 2.1. Cells, Plasmids, Antibodies and Reagents

The origin of Huh-7 [29], Huh-7.5.1 [29], Huh-7.5.1 subclone 2 (Huh-7.5.1c2) [15] and HEK-293T [30] cells have been described previously. All cells were maintained in Dulbecco’s modified Eagle’s medium (DMEM) (Cellgro; Mediatech, Herndon, VA, USA) supplemented with 10% fetal calf serum (FCS) (Cellgro), 10 mM HEPES (Invitrogen, Carlsbad, CA, USA), 100 units/mL penicillin, 100 mg/mL streptomycin, and 2 mM L-glutamine (Invitrogen) in 5% CO_2_ at 37 °C. The sub-genomic and full-length JFH-1 stable replicon Huh-7 cell lines were cultured in medium supplemented with 400 or 200 µg/mL of G418, respectively, as described previously [15]. The JFH-1 genome-containing plasmid has been previously described [31]. The JFH-1 Rluc/SGR wt or JFH-1 Rluc/SGR GND plasmids carry bicistronic constructs containing the luciferase reporter gene in the first cistron and wild-type (wt) or replication-deficient (encoding a GDD-to-GND mutation in NS5B) JFH-1 sub-genomic replicon in the second cistron, respectively [32]. The rabbit polyclonal antibody for the detection of cellular erlin-2 protein was in-house generated and affinity purified against the full-length immunogen [27]. The rabbit polyclonal antibody HPA011252 against erlin-1 protein was obtained from Sigma-Aldrich (St. Louis, MO, USA). The recombinant human IgG anti-E2, the mouse monoclonal 9E10 anti-NS5A and the rabbit polyclonal MS5 anti-NS5A antibodies were kindly provided by M. Law (Scripps Research, La Jolla, CA, USA), C. M. Rice (Rockefeller University, New York, NY, USA) and M. Houghton (University of Alberta, Edmonton, AB, Canada), respectively. The monoclonal mouse antibodies against EEA1, HCV core (clone C7-50) and NS3 (clone 2E3) proteins were obtained from BD Transduction Laboratories (Franklin Lakes, NJ), Santa Cruz Biotechnology (Santa Cruz, CA, USA) and BioFront Technologies (Tallahassee, FL, USA), respectively. The HCV RNA replication inhibitor 2′-C-methyladenosine (2 mAd) was used at 10 μM final concentration and was a gift from W. Zhong (Gilead Sciences, Foster City, CA, USA). The 3-[4,5-dimethylthiazol-2-yl]-2,5-diphenyltetrazolium bromide (MTT) was purchased from Sigma Aldrich. Protease and phosphatase inhibitors were purchased from Roche (Indianapolis, IN, USA).

### 2.2. Silencing of Erlin Proteins by siRNA Transfection

siRNAs targeting human *ERLIN1* (siErlin 1.5: CCACAAATAGGAGCAGCAT [27]) or *ERLIN2* (siErlin 2.3: GCCTCTCCGGTACTAACAT [27]) individually, or *ERLIN1* and *ERLIN2* simultaneously (siErlin 1&2: AGAAGCAATGGCCTGGTAC [27]), and the non-targeting control siRNA (siCtrol: ACTGTCACAAGTACCTACA [24]), as well as the siRNA targeting HCV genome (siHCV: ACCTCAAAGAAAAACCAAA [17]) were all previously described. All the siRNAs were purchased from Integrated DNA Technologies (IDT, San Diego, CA, USA). Typically, Huh-7 cells were plated at a density of 1 × 10^5^ cells per well on a 6-well plate. 24 h later cells were transfected with 20 pmol of the corresponding siRNA per well using Dharmafect 4 transfection reagent following manufacturer’s instructions (GE Healthcare Dharmacon Inc, Pittsburgh, PA, USA). In brief, 20 pmol of siRNA was added into 200 μL of OptiMEM containing tube and 1 μL of Dharmafect 4 reagent was added into a second tube containing another 200 μL of OptiMEM. These mixtures were incubated for 5 min at room temperature before they were mixed together and incubated for another 20 min at room temperature. During the incubation time the culture medium of the Huh-7 cells was discarded and 1.6 mL of complete DMEM medium was added to each well. After 20 min of incubation the siRNA-Dharmafect complexes-containing medium (400 μL) was added to the corresponding wells. Fresh medium was applied after overnight incubation and the cells were assayed at the indicated times after transfection as indicated in each experiment. Cell viability was determined by evaluating cell biomass at different times after siRNA transfection by crystal violet staining and colorimetry at 570 nm, as previously described [33]. As a complementary approach, the cytotoxic effect of siRNAs was evaluated by quantifying the mitochondrial activity of siRNA-transfected cells (in MTT assays) and by determining the cellular respiration capacity. The cellular respiration capacity was calculated as the mitochondrial activity relative to the total protein content of the cells, measured by BCA in wells that were transfected in parallel.

### 2.3. Preparation of Viral Stocks and Infections

The original JFH-1 virus was generated by transfection of an in vitro transcribed full-length JFH-1 HCV RNA into Huh-7 cells and viral stocks were produced by inoculation of fresh Huh-7 cells at a multiplicity of infection (moi) of 0.01 as described [29]. Production of high titer cell culture adapted JFH-1 day 183 virus (D183) [34] was achieved by inoculation of highly susceptible Huh-7.5.1c2 at low moi. These D183 virus stocks were used in all low and high moi infection experiments (moi = 0.2 or 3) as described previously [15]. Intracellular infectious HCV particles were obtained by 5 freeze-thaw cycles of infected cells as described [35]. The infectivity titers present in culture supernatants and cell extracts were determined by end-point dilution and fluorescence focus forming unit (FFU) assay in Huh-7.5.1 cells, as described previously [36].

### 2.4. Analysis of HCV Cell Entry Using HCVpp

Retroviral particles pseudotyped with the JFH-1 E1 and E2 envelope proteins or the VSV G glycoprotein, as control, were produced in HEK293T cells [37]. For HCV entry experiments, Huh-7 cells (2 × 10^4^ cells) were plated on 48-well plates and twenty hours later they were transfected with the indicated siRNAs as described above. Thirty-six hours later, cells were inoculated with pseudotyped retroviral particles (HCVpp or VSVpp) diluted to produce similar luciferase activity levels. Forty-eight hours later, cells were lysed and luciferase activity was detected and quantitated in a luminometer using a commercial kit (Luciferase Assay Kit; Promega, Madison, WI, USA). Luciferase results were normalized to cell density and relative infection values were calculated as a percentage of cells transfected with control siRNA. In parallel, cultures transfected with siRNAs were used: (i) to analyze knockdown efficiency by Western-blotting (WB) and (ii) to confirm the reduced susceptibility of erlin-1-deficient cells to HCV infection.

### 2.5. In Vitro Transcription and HCV RNA Transfection

The subgenomic JFH-1 replicon plasmid bearing a luciferase reporter gene (JFH-1 Rluc/SGR wt) and the corresponding replication-deficient construct (JFH-1 Rluc/SGR GND) have been described previously [32]. After digestion with XbaI restriction enzyme the linearized plasmids were in vitro transcribed using the T7 MEGAscript (Ambion, Austin, TX, USA) kit following the manufacturer’s instructions. siRNAs targeting the indicated genes were transfected into Huh-7 cells as described above. Twenty hours later the cells were trypsinized and cells were seeded in wells of 12-well plate at a density of 1 × 10^5^ cells per well. The following day *in vitro* transcribed subgenomic HCV RNAs were introduced into those cells by transfection using TransIT mRNA Transfection Kit (Mirus Bio LLC, Madison, WI, USA) as recommended by the manufacturer. At the indicated time points the cells were lysed. An aliquot of each lysate was used to quantitate the luciferase activity in a luminometer using a commercial kit (Promega). Another aliquot from each lysate obtained at six hours post-transfection was used to quantitate the HCV RNA levels by reverse transcription real-time quantitative PCR (RT-qPCR) as described below. The HCV RNA values were used to control for differences in the transfection efficiency between samples and were used to normalize the luciferase values. Luciferase results were normalized to cell density at each time point and the relative values were calculated as a percentage of cells transfected with control siRNA. In parallel, cultures transfected with siRNAs were used: (i) to analyze knockdown efficiency by WB and (ii) to confirm the reduced susceptibility of erlin-1-deficient cells to HCV infection.

### 2.6. siRNA Transfection Experiments in Acutely HCV Infected Cells

Huh-7 cells were transfected with the indicated siRNAs as described above. Thirty-six hours later, transfected cells were infected with JFH-1 D183 virus at low or high moi (0.2 or 3, respectively) as described before [15]. Five hours after HCV inoculation, cells were washed twice with PBS and medium was replaced. At the indicated times after infection supernatants were collected and extracellular infectivity was determined by end-point dilution in Huh-7.5.1 cell line. Cell extracts were harvested as indicated above and the intracellular infectivity, HCV RNA, and viral proteins were analyzed by titration, RT-qPCR and WB, respectively, as described [38]. Erlin protein down-regulation efficiency was confirmed by WB and densitometry analysis at the time post-infection indicated in each figure legend.

### 2.7. siRNA Transfection Experiments in Persistently Infected Cells

Huh-7 cells were infected with JFH-1 virus at a low moi = 0.01 as described [29]. Infected cells were passaged for 3 weeks before siRNAs were transfected. Three days after siRNA transfection supernatants were discarded, cells were washed twice with PBS and medium was replenished. Twenty-four hours later supernatants were collected and progeny virus released during those last twenty-four hours was determined by end-point dilution in Huh-7.5.1 cells as described above. Cell extracts were harvested and the intracellular infectivity, HCV RNA and proteins and erlin protein down-regulation were analyzed by titration, RT-qPCR and WB, respectively.

### 2.8. Protein Analysis by WB

Cell extracts were prepared in RIPA buffer supplemented with protease and phosphatase inhibitors and, after protein quantitation by bicinchorinic acid (BCA) assay (Thermo Scientific, Waltham, MA, USA), equivalent amounts of total protein (typically 20–25 μg) were separated in polyacrylamide-SDS gels by electrophoresis and they were transferred to Immobilon membranes (Millipore, Billerica, MA, USA) for blotting. The membranes were blocked for 1 h at room temperature with PBS-5% milk and incubated overnight at 4 °C with the primary antibodies diluted in 1% milk-0.1% Tween 20 in PBS. Primary antibodies were used at the following dilutions: EEA1 at 1:2000; erlin-1, erlin-2 and NS3 at 1:1000; NS5A at 1:500; and Core at 1:200 dilution. After washing at least 4 times for 15 min with 0.1% Tween 20 in PBS, membranes were incubated with a 1:10,000 dilution of goat-anti-rabbit or goat-anti-mouse IgG conjugated to horseradish peroxidase prepared in 1% milk-0.1% Tween 20 in PBS. After washing at least 4 more times with 0.1% Tween 20 in PBS, membranes were developed using the SuperSignal-West Pico or Femto chemiluminescence reagents (Thermo Scientific) and autoradiography. Densitometry of non-saturated films was performed using Fiji/Image J analysis software [39] and the results, shown beneath each panel, are expressed relative to control cells using EEA1 protein expression for normalization and as loading control. Two-fold serial dilutions of siCtrol samples were used to produce standards for relative quantitation.

### 2.9. RNA Analysis

Total RNA was extracted from cells using the guanidinium isothiocyanate extraction method [29] after adding 20 µg of glycogen (Roche) per sample as a carrier. The content of HCV RNA, *ERLIN1* and *ERLIN2* mRNAs and *GAPDH* mRNA (for normalization) in each sample was quantitated in a two-step RT-qPCR assays using the High-Capacity cDNA Reverse Transcription Kit and the Maxima SYBR Green/ROX qPCR Master Mix (2X) (Thermo Scientific). 10-fold serial dilution of plasmids containing each target sequence was used to prepare the standard curves that were used with each corresponding pair of primers: HCV (5′-TCTGCGGAACCGGTGAGTA-3′ and 5′-TCAGGCAGTACCACAAGG-3′); *ERLIN1* (5′-GGGGTTGGTGGCTGTCCTGC-3′ and 5′-TAGCCTGGTCCACTGGGGCT-3′); *ERLIN2* (5′-TGTGCACACGCTTCAAGAGGTCTA-3′ and 5′-AATGACCAGCCCAGGGGCCA-3′) and *GAPDH* (5′-GAAGGTGAAGGTCGGAGTC-3′ and 5′-GAAGATGGTGATGGGATTTC-3′). Results were normalized using *GAPDH* mRNA levels and were displayed in the figures as relative values compared to control cells.

### 2.10. Confocal Analysis

Huh-7 cells were transfected with siRNAs and infected at high moi (moi = 3) with JFH-1 D183 virus as described above. The day before fixation cells were seeded in glass bottom 96-well plates (Thermo Scientific) for confocal analysis. At the indicated times after virus inoculation, cells were incubated in 4% paraformaldehyde for 20 min at room temperature, washed 3 times with PBS and processed for immunofluorescence analysis and LD staining as previously described [38]. In brief, fixed cells were first incubated for 1 h at room temperature in blocking buffer (1xPBS, 10% FBS, 3% BSA, 0.3% Triton X-100), washed 3 times with PBS and then incubated for 1 h with a mixture of primary antibodies at the appropriate concentrations that were prepared in binding buffer (1xPBS, 3% BSA, 0.3% Triton X-100). The primary antibodies used for immunofluorescence were: a purified mouse monoclonal anti-core (clone C7-50) at 1:200 dilution and a rabbit polyclonal anti-NS5A (MS5) at 1:1000 dilution. After 3 washes with PBS, cells were incubated for one more hour in binding buffer containing a mixture of fluorophore-conjugated secondary antibodies at 1:1000 dilution (Invitrogen) and Hoechst dye (Invitrogen) for nuclei staining. LD staining was performed by incubating the cells for 30 min at room temperature with the LipidTox reagent (Invitrogen) at 1:500 dilution in PBS. Images of 1024 × 1024 pixels at 8-bit gray scale were acquired with a 40× objective (pixel size 0.345 μm) on a LSM 710 confocal laser scanning microscope (Zeiss, Dublin, CA, USA). All images were taken with exactly the same settings. Fiji/ImageJ software analysis package [39] was used to quantitate the fluorescence intensity signals in each image.

### 2.11. Statistical Analysis

**Figure 1:**
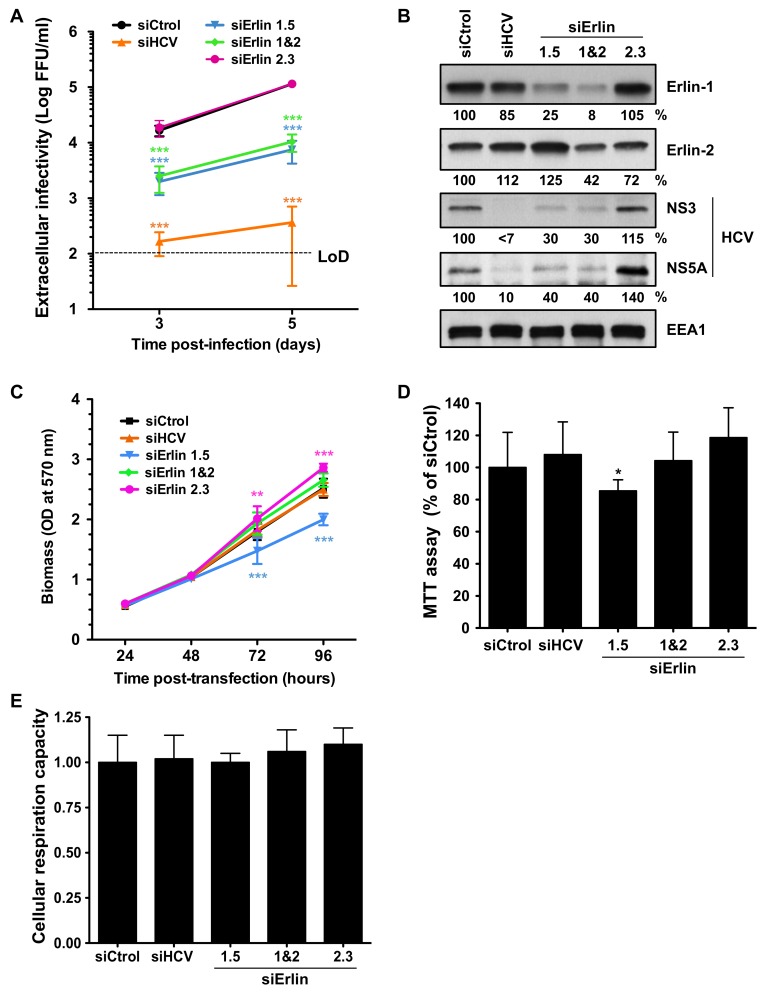
Erlin-1 protein down-regulation impairs efficient HCV infection. Huh-7 cells were transfected with individual siRNAs targeting erlin-1 (siErlin 1.5), erlin-2 (siErlin 2.3), both erlins simultaneously (siErlin 1&2), HCV (siHCV) or a non-targeting siRNA control (siCtrol) as indicated in Material and Methods section. Transfected cells were infected 36 h later with JFH-1 D183 virus at low multiplicity of infection (moi = 0.2). (**A**) The extracellular infectivity present in supernatants of infected cells was determined on days three and five post-inoculation by titration assay. Infectivity titers are represented as the FFUs per ml of supernatant in logarithmic scale and are displayed as the average and standard deviation (Mean; SD; n = 3). The horizontal black dotty line represents the limit of detection (LoD) of the assay. (**B**) Three days after virus inoculation cellular erlin-1, erlin-2 and EEA1 (as loading control) and viral NS3 and NS5A protein expression was determined in HCV-infected cell extracts by WB. Relative protein levels were determined by densitometry and are shown below each panel. The ratio of intensities of each protein and EEA1 in siCtrol-transfected cells was set as 100 and it was used to calculate the relative amount of each protein in each sample. Results in panels A and B are representative of two independent experiments, each one performed in triplicate. (**C**) Cells plated in 96-well plate format were transfected with siRNAs and the cell biomass was quantitated by crystal violet staining at different times post-transfection. Data are displayed as the average and standard deviation of two independent experiments with three replica wells per condition at each time point in each experiment (Mean; SD; n = 6). (**D**) Cells plated in 96-well plate format were transfected and infected as described above and were used to perform MTT assays three days after HCV inoculation. Data are displayed as the average and standard deviation (Mean; SD; n = 7) relative to the levels in siCtrol condition, set as 100. Results are representative of two independent experiments with seven replica wells per condition in each experiment. (**E**) The cellular respiration capacity was calculated as described in Material and Methods section. Data are displayed as the average and standard deviation (Mean; SD; n = 3) relative to that of siCtrol, set as 1. Statistical significance was determined using: Two-way ANOVA followed by a Bonferroni posttest for panels A and C (** *p* < 0.01; *** *p* < 0.001), and One-way ANOVA followed by the Dunnett’s Multiple Comparison Test for panels D and E (* *p* < 0.05).

**Figure 2:**
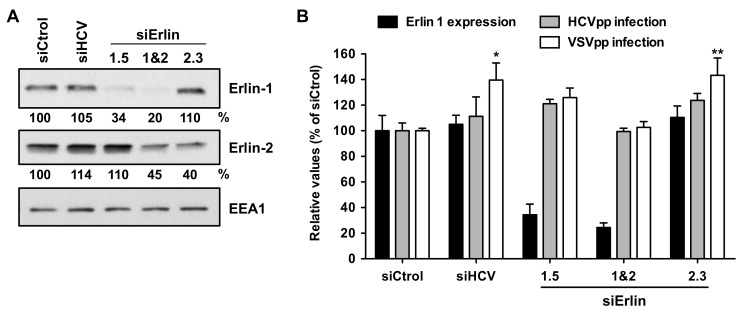
Erlin-1 protein down-regulation does not inhibit HCV cell-entry. Huh-7 cells were transfected with individual siRNAs as described in Material and Methods. (**A**) 36 h later one set of wells was harvested for erlin protein analysis by WB. Relative protein expression levels were determined by densitometry as described in the legend of Figure 1 and they are shown below each panel. (**B**) Another set of wells was inoculated with HCVpp (grey bars) or particles harboring the VSV-G glycoprotein (VSVpp; white bars) as control. 48 h after inoculation the intracellular luciferase activity was measured. Results are displayed as percentage of the luciferase activity present in siCtrol-transfected cell extracts for each virus particle. Data are displayed as the average and standard deviation (Mean; SD; n = 3). The luciferase values (units per well) in siCtrol-transfected cell extracts were 9 × 10^3^ ± 5.1 × 10^2^ and 1.2 × 10^4^ ± 2 × 10^2^ for HCVpp and VSVpp, respectively. These results are representative of two independent experiments, each one performed in triplicate. The average erlin-1 protein expression level of the two experiments is shown in black bars. One-way ANOVA followed by Dunnett’s Multiple Comparison Test analysis of the HCVpp data did not show any statistically significant effect on the luciferase levels among different siRNAs. Instead, the entry of VSVpp was significantly higher in siHCV- and siErlin 2.3-transfected cells (* *p* < 0.05; ** *p* < 0.01).

**Figure 3:**
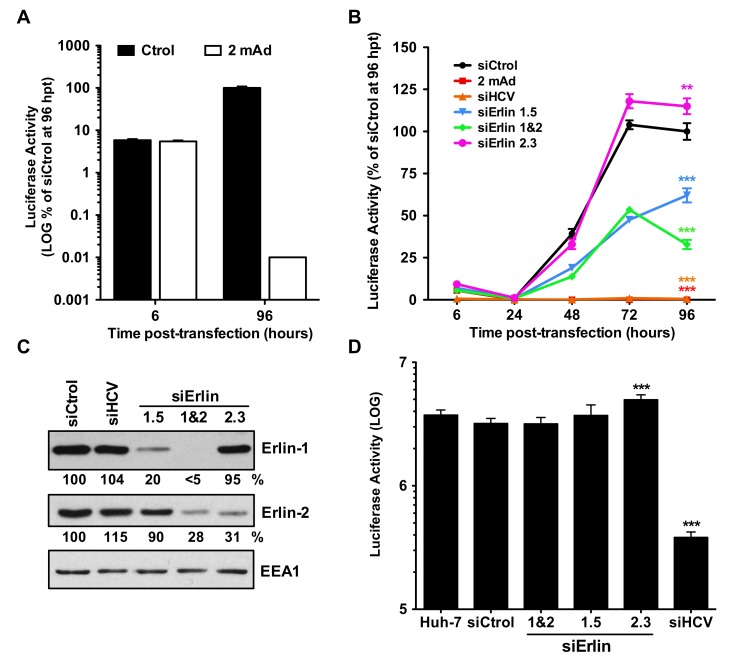
Erlin-1 protein down-regulation impairs the initiation of HCV RNA replication without affecting HCV IRES-dependent translation. Huh-7 cells were transfected with individual siRNAs as described in Material and Methods. 48 h later cells were transfected with in vitro transcribed bicistronic renilla luciferase (Rluc)-containing wt or replication-deficient (GND mutant) sub-genomic (SGR) HCV RNAs. As control for the inhibition of HCV RNA replication process, transfected cells were treated with the HCV polymerase inhibitor 2′-C-methyladenosine (2 mAd). (**A**) Quantitation of renilla luciferase activity derived from Rluc/SGR wt RNA in the presence (white bars) or absence (black bars) of 2 mAd at early (6 h) and late (96 h) time points. Data are displayed as the average and standard deviation (Mean; SD; n = 3) of luciferase values relative to that of control cells at 96 h post-transfection (hpt), that was set as 100. (**B**) Time course of renilla luciferase activity derived from RLuc/SGR wt RNA in siRNA-transfected cells. For each independent experiment, Rluc activity was normalized to cell density and was displayed as a percentage of that determined in siCtrol-transfected cell extracts at 96 hpt. Data shown are averages and standard deviation of two independent experiments, each one performed in triplicate (Mean; SD; n = 6). The luciferase activity in siCtrol-transfected cell extracts at 96 h post-transfection was 1.3 × 10^7^ ± 9.1 × 10^5^ light units per well. To assess the specificity of Rluc activity, cells were treated with 2 mAd (red line) or were transfected with siRNAs targeting directly HCV RNA (orange line). Statistical analysis performed using Two-way ANOVA followed by Bonferroni posttest showed statistically significant differences in: 2 mAd-treated, siHCV-, siErlin 1.5- and siErlin 1&2-transfected cells at 48-, 72- and 96-hpt, and siErlin 2.3-transfected cells at 72- and 96-hpt compared to siCtrol-transfected cells (** *p* < 0.01; *** *p* < 0.001). (**C**) Erlin protein down-regulation was determined at the time of Rluc/SGR RNA transfection by WB and densitometry (shown below each panel) as described in the legend of Figure 1. (**D**) Luciferase activity of replication-deficient (GND mutant) Rluc/SGR RNA was determined at six hours post-transfection. For each independent experiment Rluc activity was normalized to cell density. Data shown are averages and standard deviation of two independent experiments, each one performed in triplicate (Mean; SD; n = 6). Statistical analysis performed using One-way ANOVA followed by Dunnett’s Multiple Comparison Test showed statistically significant differences in siErlin 2.3- and siHCV-transfected cells compared to siCtrol-transfected cells (*** *p* < 0.001). No differences were observed in erlin-1 downregulated cells compared to siCtrol-transfected cells.

**Figure 4:**
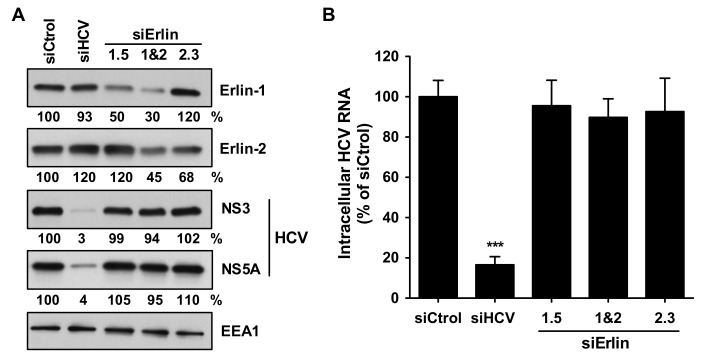
Erlin-1 protein down-regulation does not interfere with ongoing HCV RNA replication. JFH-1 sub-genomic replicon (SGR) bearing Huh-7 cells were transfected with siRNAs as described in Material and Methods. Three days after transfection cells were harvested for analysis. (**A**) Cellular erlin-1, erlin-2 and EEA1, and HCV NS3 and NS5A protein accumulation were determined by WB and densitometry (shown below each panel) as described in the legend of Figure 1. (**B**) The intracellular HCV RNA content was quantitated in infected cell extracts three days after siRNA transfection by RT-qPCR. Data were normalized relative to *GAPDH* mRNA levels in the same samples and are displayed as percentage of the HCV RNA present in siCtrol-transfected cells. The HCV RNA content in siCtrol-transfected cell extracts was 1.4 × 10^6^ ± 1.2 × 10^5^ copies per μg of total RNA. Data shown are averages of three independent experiments, each one performed in triplicate (Mean; SD; n = 9). Only the HCV RNA level in siHCV-transfected cells was significantly lower than that in siCtrol-transfected cells as determined by One-way ANOVA followed by Dunnett’s Multiple Comparison Test (*** *p* < 0.001).

**Figure 5:**
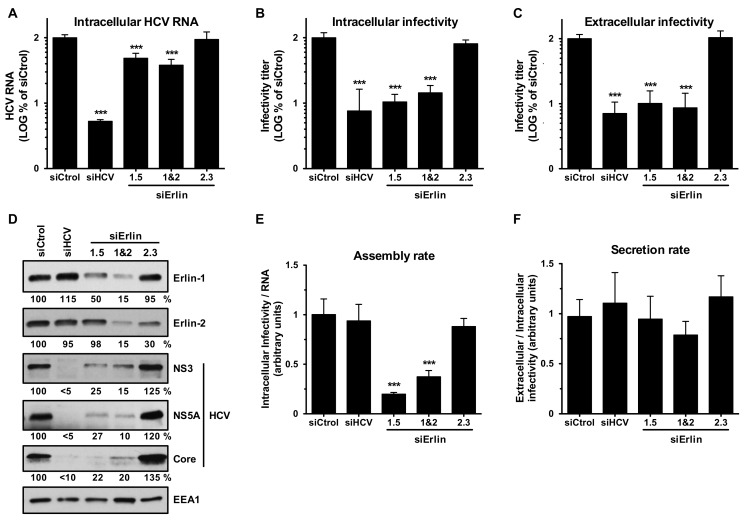
Erlin-1 protein down-regulation interferes with HCV in single-cycle infection experiments. Huh-7 cells were transfected with siRNAs as described in Material and Methods. 36 h later transfected cells were inoculated with JFH-1 D183 virus at high multiplicity of infection (moi = 3). 48 h after virus inoculation cellular extracts were prepared for intracellular RNA (**A**), infectivity (**B**) and protein (**D**) analysis and supernatants were collected for extracellular infectivity (**C**) analysis. Assembly (**E**) and secretion (**F**) rates were calculated using data from panels A, B and C. The RNA and infectivity results are displayed as percentage of the levels in siCtrol-transfected cells. The HCV RNA and the intracellular and extracellular infectivity levels in siCtrol-transfected cells were: 1.2 × 10^7^ ± 1.3 × 10^6^ HCV RNA copies per μg of total RNA, 3.5 × 10^4^ ± 6.1 × 10^3^ ffus per well, and 1.3 × 10^5^ ± 2 × 10^4^ ffus per ml of supernatant, respectively. Data shown are averages of three independent experiments, each one performed in triplicate (Mean; SD; n = 9). One-way ANOVA followed by Dunnett’s Multiple Comparison Test was used to determine the statistical significance (*** *p* < 0.001).

**Figure 6:**
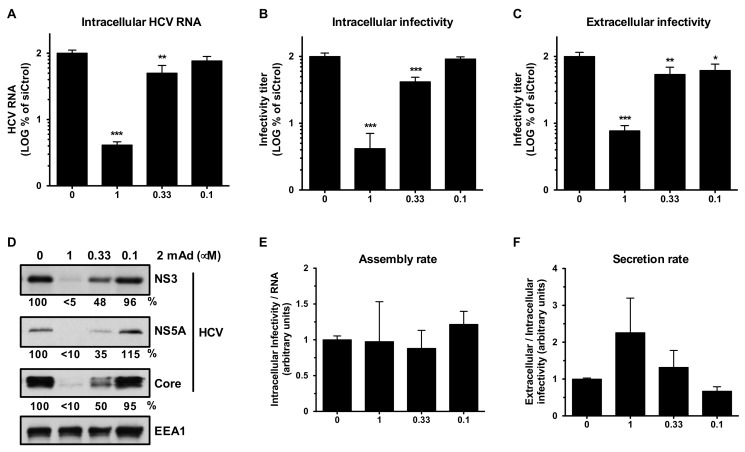
Dose-dependent reduction on HCV parameters upon treatment with a replication inhibitor. Huh-7 cells were inoculated with JFH-1 D183 virus at high multiplicity of infection (moi = 3) in the absence or presence of increasing amounts (0, 0.1, 0.33 and 1 μM) of the HCV polymerase inhibitor 2 mAd. 48 h later supernatants were collected, cell extracts were harvested and samples were subjected to the same analysis described in Figure 5. Note the proportional and dose response reduction of all viral parameters (RNA (**A**), infectivities (**B**,**C**) and protein (**D**)) in 2 mAd-treated cells and the absence of any significant effect in the assembly (**E**) and secretion (**F**) rates. The HCV RNA and the intracellular and extracellular infectivity levels in untreated cells were: 2 × 10^7^ ± 2 × 10^6^ HCV RNA copies per μg of total RNA, 2 × 10^4^ ± 2.5 × 10^3^ ffus per well, and 1.6 × 10^5^ ± 2.5 × 10^4^ ffus per ml of supernatant, respectively. Data shown are averages of two independent experiments, each one performed in duplicate (Mean; SD; n = 4). One-way ANOVA followed by Dunnett´s Multiple Comparison Test analysis was used for statistical significance analysis (* *p* < 0.05; ** *p* < 0.01; *** *p* < 0.001).

**Figure 7:**
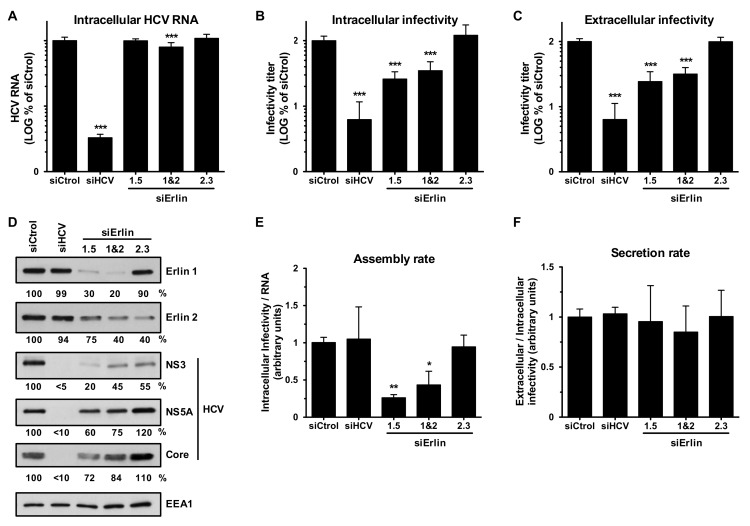
Erlin-1 protein down-regulation impairs infectious virus production in an ongoing HCV infection cell culture system. Persistently infected Huh-7 cells were transfected with siRNAs as described in Material and Methods. Four days after siRNA transfection cellular extracts were prepared for intracellular RNA (**A**), infectivity (**B**) and protein (**D**) analysis and supernatants were collected for extracellular infectivity (**C**) analysis. Assembly (**E**) and secretion (**F**) rates were calculated using data from panels A, B and C. The RNA and infectivity results are displayed as percentage of the levels in siCtrol-transfected cells. The HCV RNA and the intracellular and extracellular infectivity levels in siCtrol-transfected cells were: 5.8 × 10^5^ ± 7 × 10^4^ HCV RNA copies per μg of total RNA, 2.3 × 10^3^ ± 2.8 × 10^2^ ffus per well, and 1 × 10^4^ ± 1.1 × 10^3^ ffus per mL of supernatant, respectively. Data shown are averages of three independent experiments, each one performed in triplicate (Mean; SD; n = 9). One-way ANOVA followed by Dunnett’s Multiple Comparison Test was used to determine the statistical significance (* *p* < 0.05; ** *p* < 0.01; *** *p* < 0.001).

**Figure 8:**
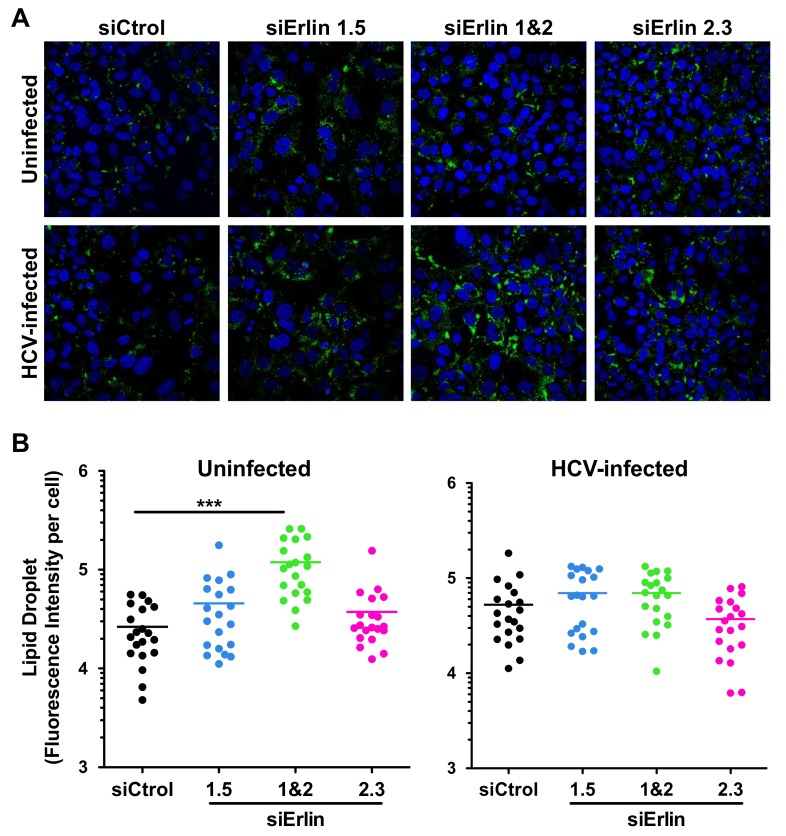
Erlin-1 protein down-regulation increases LD content in Huh-7 cells. siRNA-transfected Huh-7 cells were inoculated with JFH-1 D183 virus at high multiplicity of infection (moi = 3) and the intracellular LD content was analyzed 48 h after infection as described in Material and Methods. (**A**) Representative confocal image sections of intracellular LD accumulation (in green) in siRNA-transfected HCV-infected and uninfected cells. Nuclei (in blue) were counterstained with Hoechst dye. (**B**) Quantitation of the total LD fluorescence intensity signal per cell from confocal image sections. ImageJ software was used to quantitate the total LD fluorescence intensity signal in twenty individual cells of each condition. Each dot in the graph represents the LD fluorescence intensity of an individual cell and the horizontal lines show the average LD fluorescence intensity in each group of data. One-way ANOVA followed by Dunnett’s Multiple Comparison Test was used to determine the statistical significance (*** *p* < 0.001).

**Figure 9:**
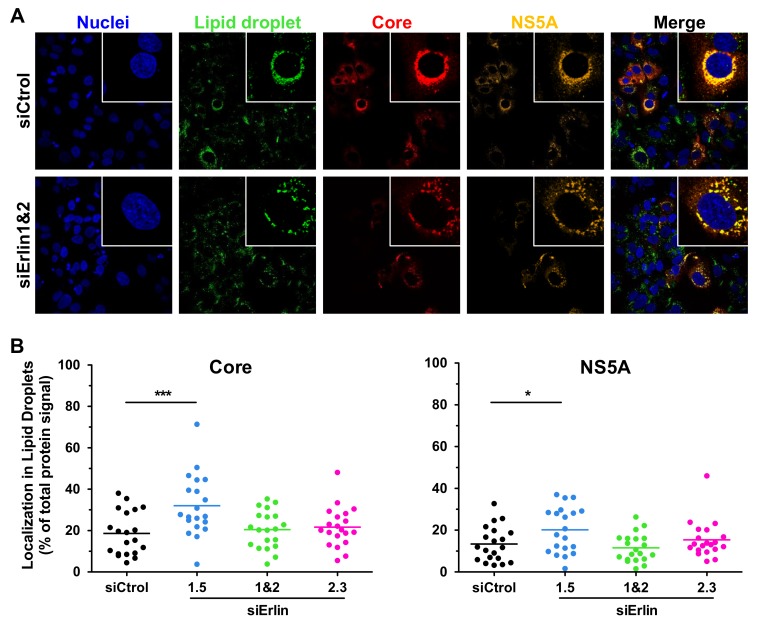
Erlin-1 protein down-regulation does not impair the localization of HCV core and NS5A proteins surrounding LDs. siRNA-transfected Huh-7 cells were inoculated with JFH-1 D183 virus at high multiplicity of infection (moi = 3) and the intracellular HCV protein and LD content was analyzed as described in Material and Methods. (**A**) Representative confocal images of the intracellular localization of HCV core (in red) and NS5A (in orange) proteins surrounding LDs (in green) in *ERLIN* down-regulated (siErlin 1&2) and control (siCtrol) HCV-infected cells. Nuclei (in blue) were counterstained with Hoechst dye. The panels on the right side show merged images of the four channels of each image. White boxes in the upper right side of each panel show zoom-in images of single cells for a more detailed observation. (**B**) The fluorescence intensity signal of core and NS5A proteins associated to LDs was quantitated in twenty randomly selected HCV-infected cells of each condition. Each dot in the graph represents the percentage of the fluorescence intensity signal of core or NS5A protein associated to LDs in a given cell, while the horizontal lines show the average of all data points in each group. One-way ANOVA followed by Dunnett’s Multiple Comparison Test was used to determine the statistical significance (* *p* < 0.05; *** *p* < 0.001).

GraphPad Prism v.5.0a software (GraphPad Software, San Diego, CA, USA) was used to analyze the data, prepare the graphs and perform all statistical analysis. All experimental results (quantitative RT-qPCR analysis, MTT assays, titration assays, luciferase activity assays, and quantitative fluorescence intensity analysis) were displayed in the graphs as the mean ± standard deviation (S.D.). The normal distribution of data was confirmed using Kolmogorov-Smirnov test in experiments with sample sizes n ≥ 6. Experiments where normality test could not be performed because of sample size limitations (n = 3) normal distribution of the data was assumed. The differences among means between multiple (more than two) groups were analyzed by One-way ANOVA or Two-way ANOVA followed by Dunnett’s or Bonferroni’s Multiple Comparison Test as indicated in each figure legend. One-way ANOVA was used for single variable analysis in Figure 1D,E, Figure 2, Figure 3D and Figure 4, Figure 5, Figure 6, Figure 7, Figure 8 and Figure 9, while Two-way ANOVA was used in kinetic experiments for two variable analysis in Figure 1A,C and Figure 3B. The statistical significance was set as: * *p* < 0.05; ** *p* < 0.01; *** *p* < 0.001.

## 3. Results

### 3.1. Erlin-1 Protein Is a Host Factor Required for Efficient HCV Infection

To investigate the potential role of erlin-1 and erlin-2 proteins in the HCV lifecycle we took advantage of siRNAs that have being previously validated and used to characterize the cellular functions of erlin proteins [24,27]. We transfected Huh-7 cells with siRNAs targeting specifically erlin-1 (i.e., siErlin 1.5) or erlin-2 (i.e., siErlin 2.3) individually, or siRNAs targeting both erlin proteins simultaneously (siErlin 1&2), siRNAs targeting HCV as a positive control (siHCV) and a non-targeting siRNA as a negative control (siCtrol). siRNA transfected cells were inoculated at low multiplicity of infection (moi = 0.2) with a cell culture-adapted HCV virus (D183v) [34] and their susceptibility to infection was assessed by measuring virus production three and five days later. Analysis of the supernatants of erlin-1 down-regulated HCV-infected cells showed ~ 7–10 fold reduction of progeny virus production compared to non-targeting control siRNA-transfected cells that were infected in parallel (Figure 1A).

Remarkably, erlin-2 protein down-regulation did not reduce HCV virus production. As expected the HCV-targeting siRNA prevented the production of infectious HCV virus. Consistent with the effect on virus production, the accumulation of intracellular HCV proteins (e.g., NS3 and NS5A) was also reduced in erlin-1, but not in erlin-2, down-regulated cells (Figure 1B). Despite a small defect in the cellular proliferation rate observed in siErlin 1.5-transfected cells (Figure 1C,D), the cellular respiration capacity of the different siRNA-transfected cells was comparable to that of control cells (Figure 1E). Collectively, these results suggest that the effects observed in erlin-1 down-regulated cells (in siErlin 1.5 and siErlin 1&2) are not due to a reduction in cell viability and most probably reflect a specific requirement of erlin-1 protein for efficient HCV infection.

### 3.2. Erlin-1 Protein Is Not Required for HCV Cell-Entry

The multiple cycle infection experiments described above suggested that erlin-1 protein plays an important role in the HCV lifecycle. Next, we set out to determine the step in the virus life cycle in which erlin-1 protein was required. Many aspects of HCV cell entry have been studied using HCV-pseudotyped retroviral vectors. Therefore, we used retroviral particles pseudotyped with the JFH-1 envelope glycoproteins E1 and E2 (HCVpp) or the vesicular stomatitis virus protein G (VSVpp), as control, to determine their ability to infect erlin-1 down-regulated cells. siRNA-transfected Huh-7 cells were infected with the corresponding retroviral particles and the intracellular luciferase activity was measured forty-height hours later. Down-regulation efficiency was verified in parallel cultures by WB (Figure 2A). As shown in Figure 2B, transfection of *ERLIN1*-targeting siRNAs had no effect on viral entry efficiency despite strong reduction in erlin-1 protein expression achieved in those cells. As expected *ERLIN2*- and HCV-specific siRNAs had no effect on the HCVpp entry process. These results suggest that reduced erlin-1 protein expression does not affect HCV cell entry.

### 3.3. Erlin-1 Protein Down-Regulation Impairs the Establishment of HCV RNA Replication but Does Not Affect Primary Translation or Maintenance Of Replication

To test whether erlin-1 protein regulates early steps downstream HCV entry such as the primary translation of the incoming RNA or the establishment of RNA replication, we used a highly sensitive approach based on the transfection of an in vitro transcribed JFH-1 subgenomic RNA bearing a luciferase reporter gene (JFH-1 Rluc/SGR RNA). In this system the luciferase activity measured at early time points after transfection (i.e., at six hours post-transfection) derives exclusively from the translation of transfected RNA, while the accumulation of luciferase at later time points (e.g., at 48, 72 or 96 h post-transfection) is the result of both translation and RNA replication processes. Consistent with this, luciferase activity derived from JFH-1 Rluc/SGR wt RNA was equivalent in the presence (white bars) or absence (black bars) of the HCV polymerase inhibitor 2′-C-methyladenosine (2 mAd) six hours after transfection (Figure 3A). However, it was clearly reduced in 2 mAd-treated cells at 96 h post-transfection.

Luciferase activity measured at early time points (6 and 24 h post-transfection) was similar in all siRNA-transfected cell lines, suggesting that primary translation is not affected by the levels of erlin-1 or erlin-2 protein expression (Figure 3B). Interestingly, the luciferase activity in erlin-1 down-regulated cells (siErlin 1.5- and 1&2-transfected cells) was around 50% lower than that of control cells at 48 and 72 h post-transfection. Moreover, the luciferase activity measured at 96 h time point showed greater differences in siErlin 1&2-transfected cells (30% of siCtrol) than in siErlin 1.5-transfected cells (60% of siCtrol), correlating with erlin-1 down-regulation efficiencies achieved by the corresponding siRNAs (Figure 3C). These results suggest that erlin-1 protein regulates the establishment of HCV RNA replication without affecting the primary translation. Supporting this notion, luciferase activity measured six hours after the transfection of a replication deficient JFH-1 RLuc/SGR GND mutant RNA was comparable in erlin-1 down-regulated cells and control cells (Figure 3D). Altogether, these data suggest that erlin-1 protein regulates the initiation of RNA replication without affecting the translation of incoming HCV RNA.

Next, we analyzed the effect of erlin-1 protein down-regulation on the maintenance of HCV RNA replication in the stably replicating JFH-1 subgenomic replicon cells (SGR cells). SGR cells were transfected with the indicated siRNAs and three days later the intracellular erlin protein levels and HCV protein and RNA levels were analyzed by WB and RT-qPCR, respectively. As expected, transfection of the HCV-targeting siRNA produced a significant reduction of HCV proteins (Figure 4A) and RNA (Figure 4B). However, no effect was observed when siRNAs targeting erlin-1 protein were transfected despite the 50–70% reduction in erlin-1 protein expression achieved. Similar results were obtained when JFH-1 full-length replicon cells were used (data not shown). These results suggest that erlin-1 protein does not regulate the ongoing HCV RNA replication process.

### 3.4. Erlin-1 Protein Down-Regulation Interferes with HCV Protein and Intracellular Infectious Virus Accumulation

Collectively the results from the experiments described above suggested that reduction of erlin-1 protein expression impairs HCV infection by inhibiting the establishment of RNA replication (Figure 3) without affecting HCV entry (Figure 2), HCV IRES dependent primary translation (Figure 3) or ongoing HCV RNA replication (Figure 4). Consistent with those results, quantitation of the intracellular HCV RNA levels 48 h after a high moi (moi = 3) infection showed a modest but statistically significant two to three-fold decrease in erlin-1 down-regulated cells compared to control cells (Figure 5A). Interestingly, the accumulation of intracellular (Figure 5B) and extracellular (Figure 5C) infectious virus was strongly reduced (around ten-fold) in those same cells. These results were confirmed with a different *ERLIN1*-targeting siRNA (siErlin 1.3 in Supplementary Figure S1). Similarly, the accumulation of HCV core, NS3 and NS5A proteins was reduced by four to ten-fold in erlin-1 down-regulated cells (Figure 5D). The disproportionate effect on the infectivity levels compared to the effect on the intracellular HCV RNA level suggested that erlin-1 protein is required not only for the establishment of RNA replication as shown in Figure 3, but also for a post-replication step in the virus lifecycle. In fact, the assembly rate of infectious virus particles (Figure 5E) but not the secretion rate (Figure 5F) was reduced in erlin-1 down-regulated cells compared the control cells. Supporting the latest, siRNAs that target directly the HCV genome (i.e., siHCV) inhibited HCV RNA accumulation and downstream HCV protein accumulation as well as intra- and extra-cellular infectious virus accumulation to the same extent (around 15–20 fold). 

Moreover, dose-dependent and parallel reductions in HCV parameters (i.e., intracellular HCV RNA, protein and infectious virus, and extracellular infectious virus) were observed when cells were infected in the presence of different doses of the HCV polymerase inhibitor 2 mAd (Figure 6). These results imply that the disproportionate effect in virus infectivity levels observed in erlin-1 down-regulated cells could not be explained solely by the modest reduction in intracellular HCV RNA accumulation suggesting an independent requirement for erlin-1 protein at a post RNA replication step.

To prove unequivocally that erlin-1 protein is a rate limiting factor for a later step in the HCV virus life cycle we took advantage of the possibility of manipulating erlin-1 protein expression after the HCV infection is fully established. This approach allowed us to avoid the effect of erlin-1 protein down-regulation in the establishment of RNA replication described above. siRNAs were transfected into persistently HCV-infected cells and four days later the intracellular HCV RNA (Figure 7A) and the intracellular (Figure 7B) and extracellular (Figure 7C) infectious virus accumulation were quantitated by RT-qPCR and titration assays, respectively. While siRNAs targeting HCV genome significantly suppressed HCV RNA and infectious virus accumulation, *ERLIN1*-targeting siRNAs reduced infectious virus production by 70–80% with a modest 10–20% reduction on HCV RNA levels. This was reflected in a lower assembly rate (Figure 7E), but not in the secretion rate (Figure 7F) in erlin-1 down-regulated cells. These results were confirmed with a different *ERLIN1*-targeting siRNA (siErlin 1.3 in Supplementary Figure S2). HCV protein analysis showed stronger defect in NS3 than in NS5A and core protein accumulation in erlin-1 down-regulated cells (Figure 7D). These results strongly suggest that erlin-1 protein regulates later event(s) that lead to infectious virus production.

### 3.5. Erlin-1 Protein Down-Regulation Increases LD Accumulation in Huh-7 Cells

We have previously described that down-regulation of erlin proteins lead to an intracellular fatty acids and cholesterol increase in Hela cells [27]. Furthermore, it is well known that HCV requires LDs for assembly of infectious virus and that HCV induces LD accumulation in infected cells [10]. Therefore, we analyzed the effect of erlin protein down-regulation on LD accumulation in HCV-infected Huh-7 cells. To do so, erlin down-regulated and control cells were infected with JFH-1 D183 virus at high moi (moi = 3) and fixed forty-eight hours later for analysis. Fixed cells were incubated with LipidTox, a reagent that stains specifically neutral lipids, and they were analyzed by confocal microscopy. As shown in Figure 8A, reduction in erlin-1 and erlin-2 protein expression produced an increase in LD accumulation compared to control cells. Quantitation of fluorescence intensity in individual cells confirmed those results and revealed a statistically significant increase in LD accumulation in cells where *ERLIN1* and *ERLIN2* were simultaneously down-regulated (i.e., siErlin 1&2) compared to that of control cells (Figure 8B). These results suggest that erlin protein down-regulation increases the intracellular accumulation of LDs not only in Hela cells, as previously described [27], but also in Huh-7 cells.

### 3.6. Erlin-1 Protein Deficiency Does Not Impair HCV Core and NS5A Protein Re-Localization to LDs

Late in the infection both core and NS5A proteins are known to be associated to LDs to facilitate virus assembly [10]. Thus, we analyzed the distribution of core and NS5A proteins in *ERLIN* downregulated cells by confocal microscopy. As shown in Figure 9A, core and NS5A proteins localized to LDs similarly in both control and *ERLIN* down-regulated cells. A quantitative analysis of the localization of core and NS5A proteins surrounding LDs (Figure 9B) confirmed that the reduced infectious virus accumulation observed in *ERLIN1*-deficient cells was not due to a mislocalization of core or NS5A proteins during infection.

## 4. Discussion

The development of an in vitro HCV infection system in 2005 [29,40,41] allowed the identification of viral proteins and cellular factors and pathways that regulate different aspects of the HCV infection (reviewed in [8,42,43]). Using this infection model, we and others have previously identified cellular factors required for initiation of RNA replication [17,32] and HCV assembly and secretion [15,38], and cellular pathways required for the induction [44,45] and evasion [36,46] of the innate immune system. In this study, we have analyzed the consequences of erlin-1 and erlin-2 protein down-regulation on the susceptibility of Huh-7 cells to HCV infection. Our data suggest a role for erlin-1 protein early and late in the HCV life cycle. 

Silencing of erlin-1 but not erlin-2 protein expression by siRNAs led to a strong reduction in the production of infectious progeny virus (Figure 1) suggesting that erlin-1 protein is required for efficient HCV infection. Single cycle infection experiments in *ERLIN1* down-regulated cells showed a modest 2–3 fold reduction in intracellular HCV RNA accumulation compared to a ~4–10 fold reduction in HCV protein accumulation and ~10-fold reduction in intracellular infectious virus accumulation (Figure 5 and Supplementary Figure S1). Further studies performed in HCV surrogate models that interrogate specific steps in the virus life cycle revealed that erlin-1 protein is required for the initiation of HCV RNA replication (Figure 3) without affecting earlier steps such as HCV cell entry (Figure 2) and HCV IRES dependent translation of the incoming RNA (Figure 3). Interestingly, *ERLIN1* silencing in subgenomic replicon cells (Figure 4) or in persistently infected cells (Figure 7) had no effect on the steady-state level of HCV RNA but revealed differences in the steady-state levels of HCV proteins. Collectively, these results suggested that erlin-1 protein plays a role in the initiation of RNA replication but it seems to be dispensable for the maintenance of RNA replication once it has been established. The formation of the first viral replication complexes and the initiation of RNA replication are thought to take place in the presence of very low levels of viral proteins. Therefore, it is conceivable that the initiation step is more likely to be dependent on host factors than later steps when the accumulation of viral factors required for RNA replication is higher and the virus has induced a more favorable environment for replication in the cell by inducing the membranous web [47]. Our results are consistent with this notion and add erlin-1 protein to the list of factors previously reported to show a differential requirement for the initiation and maintenance of the RNA replication processes [17,38,48]. Alternatively, these results might suggest the existence of a different threshold for the requirement of erlin-1 protein in the regulation of the initiation and maintenance of RNA replication i.e., higher threshold for initiation than for maintenance.

Analysis of virus accumulation in acutely (Figure 5 and Supplementary Figure S1) and persistently (Figure 7 and Supplementary Figure S2) infected *ERLIN1*-deficient cells showed a strong reduction in the production of both intracellular and extracellular infectious virus. Biophysical analysis of infectious virus particles produced in *ERLIN1*-deficient cells did not reveal any difference in terms of their specific infectivity (FFU per RNA copies) or density profiles compared to the virus produced in control cells (data not shown) suggesting that *ERLIN1*-deficient cells produce qualitatively normal but quantitatively less infectious virus than *ERLIN1*-sufficient cells.

The disproportionate reduction in virus production compared to the decrease in HCV RNA levels suggested that erlin-1 protein regulates not only early steps leading to RNA and protein accumulation, but also later steps affecting virus production. In fact, direct targeting of RNA replication by an HCV polymerase inhibitor led to a proportional decrease of HCV RNA, protein and infectious virus, suggesting that the strong inhibition of virus production observed in *ERLIN1* down-regulated cells could not be solely explained by the modest reduction in the HCV RNA levels (Figure 6). As mentioned above, the magnitude of HCV NS3, NS5A and core protein reduction was disproportionate to the magnitude of HCV RNA reduction suggesting that *ERLIN1* down-regulation affects the steady-state levels of HCV proteins. The HCV-IRES translation results indicated that the translation of HCV RNA itself is not inhibited in *ERLIN1*-deficient cells (Figure 3) suggesting that the effect is likely to be at the level of protein stability or degradation and not at the translational level. This hypothesis is very appealing since erlin proteins have been implicated in the modulation of the ERAD pathway [23,24,25,26]. In contract to the reduced HCV protein accumulation observed in erlin-1 protein deficient HCV-infected cells (Figure 5), *ERLIN1* and *ERLIN2* deficiency produces an accumulation of cellular substrates that are typically degraded through the ERAD pathway [23,24,25,26]. These opposite effects on protein homeostasis (HCV vs. cellular proteins) may suggest that cellular proteome changes specifically induced by *ERLIN1* down-regulation could indirectly be responsible, at least in part, of the effects observed in the HCV infection. Comparison of cellular proteomes of *ERLIN1*- and *ERLIN2*-deficient cells could identify cellular candidate proteins contributing to the effects observed in *ERLIN1*-deficient HCV-infected cells. 

Recruitment of viral NS5A and core proteins to LDs is a prerequisite for virus assembly [10]. Indeed, interfering with viral protein trafficking to LDs reduces virus production [13,49]. However, viral protein localization into LDs is not sufficient for virus particle assembly. For instance, a single mutation in the core protein (Y136A) of J6/JFH-1 virus has been associated with an increased accumulation of core in LD, decreased colocalization with E2 protein and reduced HCV particle assembly [50]. Confocal analysis of infected cells confirmed the reduced expression of HCV NS5A and core proteins and revealed a typical localization of these proteins surrounding LDs late in the infection, suggesting that *ERLIN1*-deficiency is affecting their abundance (Figure 5 and Figure 7) but not their recruitment to the HCV assembly factories (Figure 9). Collectively these results suggest that the reduction in HCV protein levels could be responsible for the strong inhibition of infectious virus production observed in erlin-1 down-regulated cells. Importantly, given the differential effect in HCV RNA levels and virus production, these results suggest to us the existence of different HCV protein thresholds required to maintain RNA replication and assembly processes.

Another interesting aspect of these studies is that the HCV-related effects are specific to *ERLIN1* deficiency but not to *ERLIN2* deficiency despite their high similarity and functional cellular redundancy. This difference could be interpreted as a function of protein depletion efficiency achieved by the different siRNAs. *ERLIN1*-targeting siRNAs (1.3, 1.5 and 1&2) were in general more efficient (stronger reduction and longer-lasting effect) reducing erlin-1 protein expression than *ERLIN2*-targeting siRNAs (2.3 and 1&2) in silencing erlin-2 protein. The reason for this could be in part the longer half-life of erlin-2 compared to erlin-1 protein and the relative abundance of those proteins in Huh-7 cells, with erlin-2 being ~2–3 times more abundant than erlin-1 protein (data not shown). Alternatively, our results might indeed reflect a specific function of erlin-1 protein that is not shared by erlin-2 protein and that would be the first function exclusively assigned to erlin-1 protein. Importantly, our results identify erlin-1 protein as a new positive modulator of HCV infection and together with the results described by Inoue et al. in the SV40 infection system [28] point to the need of investigating the role of erlin proteins in other viral infections, especially in those in which their life cycle is tightly associated with the ER e.g., dengue virus or zika virus infection.

## Supplementary Materials

The following are available online at https://www.mdpi.com/2073-4409/8/12/1555/s1, Figure S1: Erlin-1 protein down-regulation interferes with HCV in single-cycle infection experiments, Figure S2: Erlin-1 protein down-regulation impairs infectious virus production in an ongoing HCV infection cell culture system.
