# Supplementary material for: The Host Factor Erlin-1 is Required for Efficient Hepatitis C Virus Infection
Source: Cells. 2019 Dec 2;8(12):1555. doi: 10.3390/cells8121555 (PMC6953030; doi:10.3390/cells8121555)
Supplement: Supplementary file 1 [file cells-08-01555-s001.pdf]

Supplementary Information

Supplementary Materials and Methods

Silencing of erlin proteins by siRNA transfection

siRNAs targeting human *ERLIN1* (siErlin 1.3: CCTTATAGCTGCACAGAAA and siErlin 1.5: CCACAAATAGGAGCAGCAT [27]) individually, or *ERLIN1* and *ERLIN2* simultaneously (siErlin 1&2: AGAAGCAATGGCCTGGTAC [27]), and the non-targeting control siRNA (siCtrol: ACTGTCACAAGTACCTACA [24]), were transfected and then infected as described in Materials and Methods section in the main text.

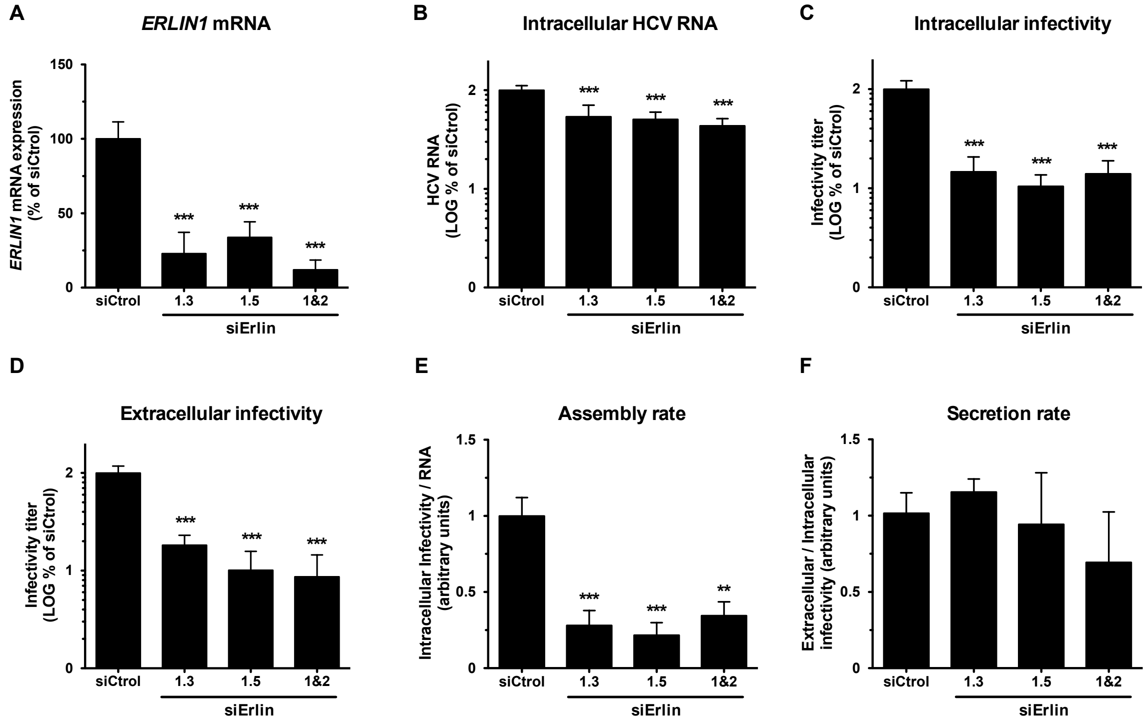

Figure S1. Erlin-1 protein down-regulation interferes with HCV in single-cycle infection experiments. Huh-7 cells were transfected with three different *ERLIN1*-targeting siRNAs as described in Material and Methods. 36 hours later transfected cells were inoculated with JFH-1 D183 virus at high multiplicity of infection (moi = 3). 48 hours after virus inoculation cellular extracts were prepared for intracellular *ERLIN1* mRNA (A), HCV RNA (B) and infectivity (C) analysis and supernatants were collected for extracellular infectivity (D) analysis. Assembly (E) and secretion (F) rates were calculated using data from panels B, C and D. The RNA and infectivity results are displayed as percentage of the levels in siCtrol-transfected cells. Data shown are averages of two independent experiments, each one performed in triplicate (Mean; SD; n=6). One-way ANOVA followed by Dunnett's Multiple Comparison Test was used to determine the statistical significance (** $P < 0.01$, *** $P < 0.001$). These data confirm the results shown in Figure 5 with an additional *ERLIN1*-targeting siRNA (i.e. siErlin 1.3).

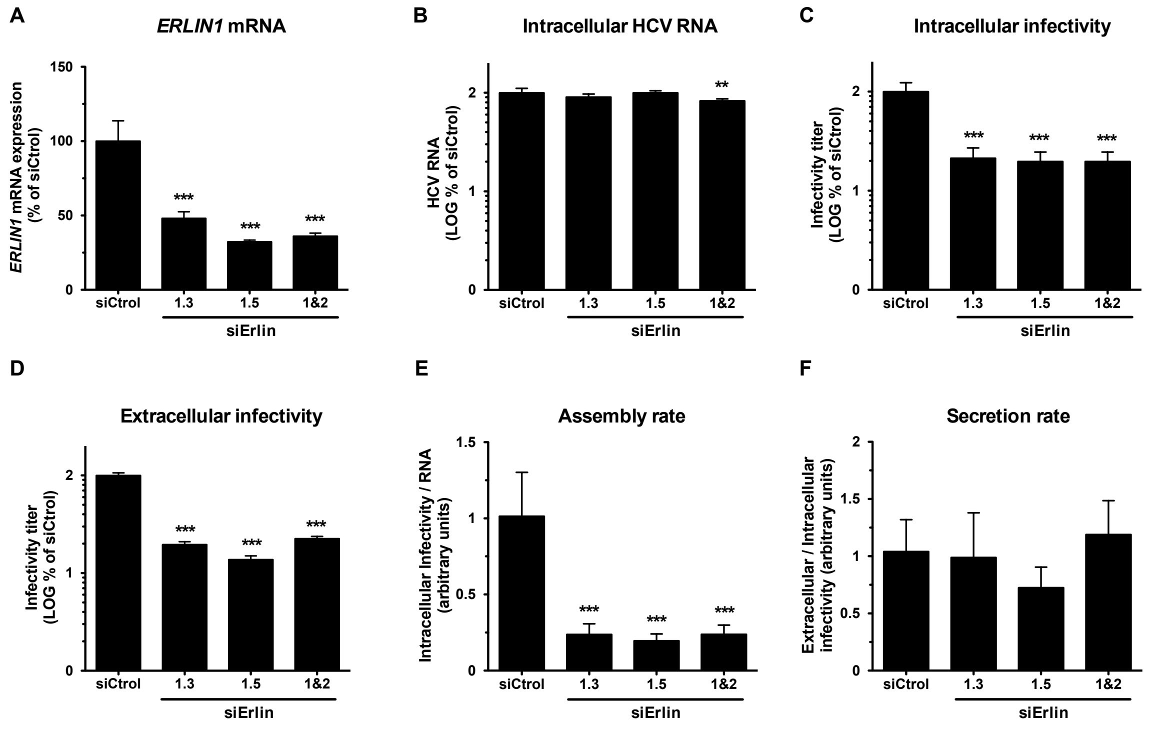

Figure 2. Erlin-1 protein down-regulation impairs infectious virus production in an ongoing HCV infection cell culture system. Persistently infected Huh-7 cells were transfected with three different *ERLIN1*-targeting siRNAs as described in Material and Methods. Four days after siRNA transfection cellular extracts were prepared for intracellular *ERLIN1* mRNA (A), HCV RNA (B) and infectivity (C) analysis and supernatants were collected for extracellular infectivity (D) analysis. Assembly (E) and secretion (F) rates were calculated using data from panels B, C and D. The RNA and infectivity results are displayed as percentage of the levels in siCtrol-transfected cells. Data shown are averages of a single experiment performed with three biological replicates (Mean; SD; n=3). One-way ANOVA followed by Dunnett's Multiple Comparison Test was used to determine the statistical significance (** $P < 0.01$; *** $P < 0.001$). These data confirm the results shown in Figure 7 with an additional *ERLIN1*-targeting siRNA (i.e. siErlin 1.3).
